# Thermally tunable add-drop filter based on valley photonic crystals for optical communications

**DOI:** 10.1515/nanoph-2024-0437

**Published:** 2024-10-28

**Authors:** Lu Sun, Xingfeng Li, Pan Hu, Hongwei Wang, Yong Zhang, Guojing Tang, Xintao He, Jianwen Dong, Yikai Su

**Affiliations:** State Key Lab of Advanced Optical Communication Systems and Networks, Department of Electronic Engineering, 12474 Shanghai Jiao Tong University, Shanghai 200240, China; State Key Laboratory of Optoelectronic Materials and Technologies, School of Physics, Sun Yat-Sen University, Guangzhou 510275, China

**Keywords:** topological photonics, valley photonic crystals, optical communications

## Abstract

Valley photonic crystals (VPCs) provide an intriguing approach to suppress backscattering losses and enable robust transport of light against sharp bends, which could be utilized to realize low-loss and small-footprint devices for on-chip optical communications. However, there are few studies on how to achieve power-efficient tunable devices based on VPCs, which are essential for implementing basic functions such as optical switching and routing. Here, we propose and experimentally demonstrate a thermally tunable add-drop filter (ADF) based on VPCs operating at telecommunication wavelengths. By leveraging the topological protection of the edge state and the distinct property of negligible scattering at sharp bends, a small footprint of 17.4 × 28.2 μm^2^ and a low insertion loss of 2.7 dB can be achieved for the proposed device. A diamond-shaped microloop resonator is designed to confine the light and enhance its interaction with the thermal field generated by the microheater, leading to a relatively low power of 23.97 mW needed for switching the output signal from one port to the other. Based on the thermally tunable ADF under the protection of band topology, robust data transmission is implemented with an ultrahigh data rate of 132 Gb/s. Our work shows great potential for developing high-performance topological photonic devices with the thermally tunable silicon-based VPCs, which offers unprecedented opportunities for realizing topologically protected and reconfigurable high-speed datalinks on a chip.

## 1 Introduction

Topology, an important concept in modern physics, has attracted considerable attention across various fields, including condensed matter physics [1], [2], [3], photonics [4], [5], [6], acoustics [7], [8], and mechanics [9], [10]. Photonic topological insulators (PTIs) are structures where photons are forbidden in the bulk but can propagate along the edge. The transport of light is protected by the band topology, which leads to the unique properties of disorder immunity and back-scattering suppression [11]. It makes PTIs a promising platform for realizing low-loss and small-footprint devices for on-chip photonic circuits. PTIs can be implemented in several ways, including gyromagnetic photonic crystals (PCs) under an applied magnetic field [12], [13], bi-anisotropic metamaterials [14], [15], coupled waveguides and microring resonators (MRRs) [16], [17], dielectric PCs with pseudo-time-reversal symmetry [18], [19], etc. Compared to these methods, valley PCs (VPCs) are more preferred since they are non-magnetic, metal-free, compact and can support long-range in-plane propagation of light [20], [21]. They can be realized by breaking the spatial inversion symmetry of the lattices in all-dielectric PCs operating at telecom wavelengths. Since their emergence, great efforts have been devoted to developing functional devices based on VPCs, such as lasers [22], routers [21], splitters [23], etc. These achievements prove that VPCs show great potential in implementing topologically protected devices with novel functions for a variety of applications such as optical communications and quantum information processing [19], [23].

Tunable or reconfigurable devices are crucial in applications such as optical signal switching and routing. Several topological photonic devices have been reported to be optically or mechanically controllable [24], [25]. However, these methods remain challenging for achieving accurate control over large-scale photonic integrated circuits. To address this issue, electrically controllable VPCs based on the thermo-optic effect of silicon have been proposed to realize thermally tunable optical switches and modulators using Mach–Zehnder interferometers (MZIs) and MRRs, respectively [26], [27]. Despite these efforts, there is still a high demand for tunable topological photonic devices with low losses and compact footprints. Thermal tuning has the advantages of easy fabrication, wide tuning range and negligible losses induced by the microheaters [28]. However, the tuning speed is on the order of microseconds which is slower than that of electrical tuning based on the plasma dispersion effect of silicon [29], [30]. Here, we propose and experimentally demonstrate a thermally tunable add-drop filter (ADF) based on silicon VPCs. The thermal effect on the edge states is theoretically investigated and exploited to design a compact and efficient device. A microloop resonator is employed to confine the light and enhance the interaction between the light and thermal fields. As a result, the fabricated device has a small footprint of 17.4 × 28.2 μm^2^ and a low insertion loss (IL) of <3 dB around 1,540 nm. An electrical power of 23.97 mW is required to switch the output optical power from the drop (through) port to the through (drop) port. Obviously, the proposed device has a lower IL than the previously reported works based on the thermally tunable VPCs [26], [27], as well as a small size and a low switching power comparable to them, which is beneficial for achieving on-chip optical communications with very high data rates. To verify the feasibility of using the topological photonic ADF in a practical communication system, a high-speed data transmission experiment is carried out with a 66-GBaud four-level pulse amplitude modulation (PAM-4) signal. The sensitivity penalty induced by the device is less than 0.7 dB compared with the optical back-to-back (OBTB) case, indicating its capability of high-capacity transmission in the telecom band. Our work reveals the possibility of implementing high-performance and tunable topological photonic devices with VPCs which may find applications in many fields ranging from ultrahigh-bit-rate communications to classical and quantum computing.

## 2 Results

### 2.1 Design of VPCs

**Figure 1:**
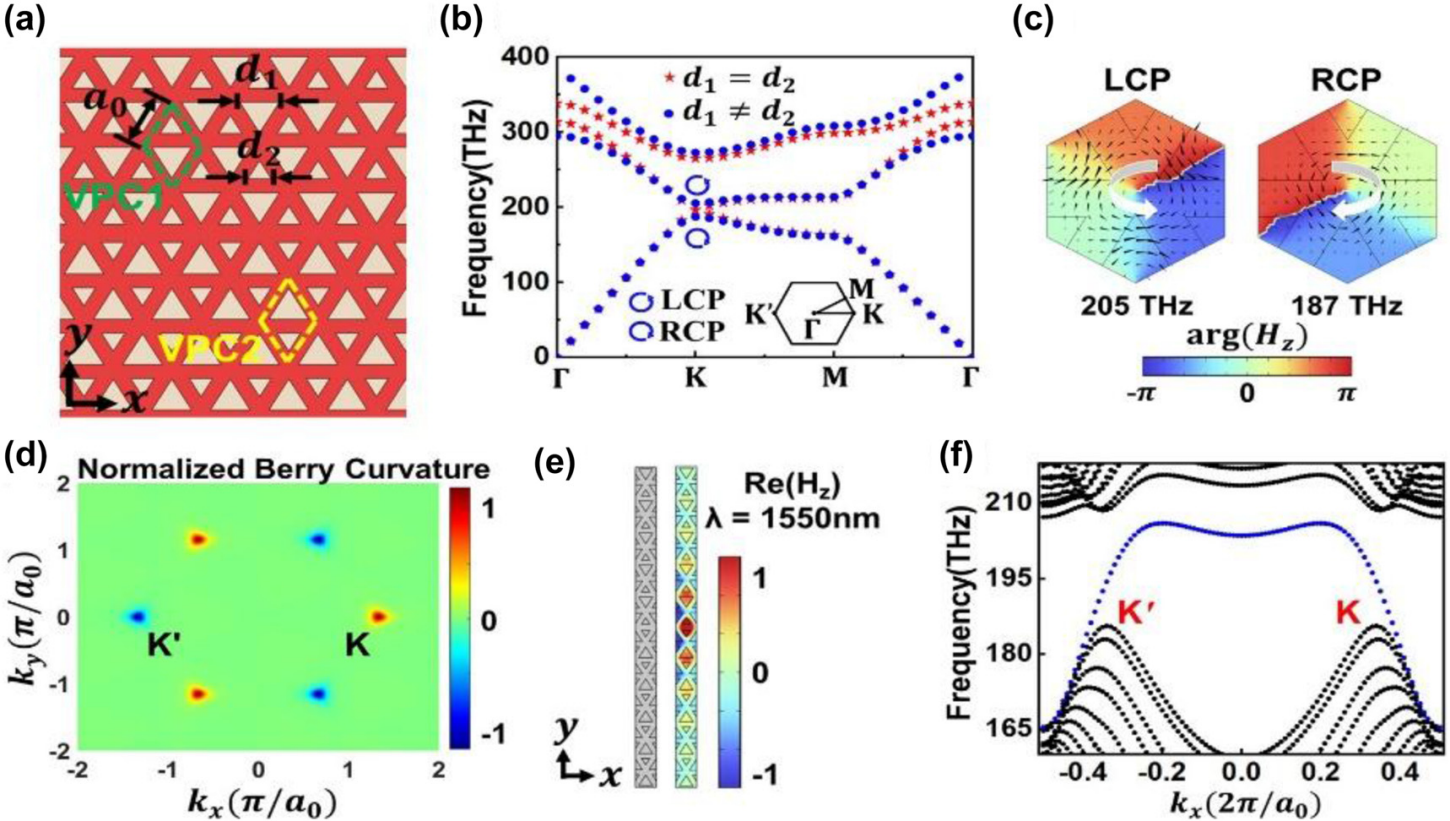
Schematic and operation principle of the VPCs. (a) Schematic of the proposed PC structure. A domain wall exists between VPC1 and VPC2 whose unit cells are shown in the green and yellow dashed rhombic boxes, respectively. The pink and grey colors represent silicon and silica, respectively. The lattice constant is *a* = 430 nm. The side lengths of the big and small triangular holes are *d*_1_ = 290 nm and *d*_2_ = 170 nm, respectively. (b) Band structure of VPC1 or VPC2 with *C*_6v_ symmetry (red stars) versus *C*_3v_ symmetry (blue dots). The inset shows the first Brillouin zone of the proposed VPCs. The LCP and RCP valley states are marked by the counterclockwise and clockwise twisted arrows, respectively. (c) Phase distributions of the *H*_*z*_ field for the *K* valley states at 187 THz and 205 THz. The black arrows indicate the direction of the Poynting power flow. (d) Normalized Berry curvature of the first band of VPC2 in the first Brillouin zone. (e) Schematic of the structure (left) and *H*_*z*_ field distribution of the edge state (right) in one period along the *x* direction. (f) Dispersion of the edge state (blue) appearing in the band gap between the first and second bulk bands (black). It corresponds to the edge mode localized on the interface between VPC1 and VPC2 as shown in (a).

Figure 1(a) shows a schematic of the VPCs fabricated on a silicon-on-insulator (SOI) platform with a 220-nm-thick top silicon layer, a 3-μm-thick buried oxide layer and a 1-μm-thick silica upper cladding layer. An interface exists between two types of VPCs, namely VPC1 and VPC2. The unit cells of VPC1 and VPC2 are shown in the green and yellow dashed rhombic boxes, respectively. Each unit cell contains two inverted equilateral triangular holes of different side lengths, i.e., *d*_1_ and *d*_2_. The lattice constant is *a*_0_ = 430 nm. Here, we focus on the transverse electric (TE)-like modes supported by the VPCs since they propagate in the *x*–*y* plane and are highly confined in the *z* direction. The band diagram of the TE-like modes in VPC1 or VPC2 is plotted in Figure 1(b). When the spatial inversion symmetry of the VPC structure is unbroken (*d*_1_ = *d*_2_ = 215 nm), the PC lattice exhibits a *C*_6v_ symmetry, featuring a Dirac cone at the *K* and *K*′ points in the momentum space, as shown by the red stars in Figure 1(b). By introducing asymmetry between the sizes of the two holes within the unit cell (*d*_1_ = 290 nm and *d*_2_ = 170 nm), the spatial inversion symmetry is broken and the lattice symmetry is reduced from *C*_6v_ to *C*_3v_. As a result, the degeneracy is lifted and a band gap (187–205 THz) opens at the *K* (*K*′) point, as illustrated by the blue dots in Figure 1(b). Figure 1(c) shows the phase profiles of the *H*_*z*_ fields, i.e., arg(*H*_*z*_), for the Bloch modes on the two lowest bands at the *K* point. The chirality of the mode can be characterized by the topological charge. It is defined as $l=\oint_{L}\nabla \left[\arg\left(H_{z}\right)\right]\cdot d\vec{s}/2\pi$ , where *L* is a closed contour surrounding the unit cell center [31]. For the *K* valley state at 205 THz, the phase decreases counterclockwise, which corresponds to a left-handed circular polarization (LCP) with *l* = −1. In contrast, the phase increases counterclockwise for the *K* valley state at 187 THz, corresponding to a right-handed circular polarization (RCP) with *l* = 1. By using the plane wave expansion (PWE) method and applying the $\vec{k}\cdot \vec{p}$ approximation, the two lowest bands can be described analytically by an effective Hamiltonian near the *K*/*K*′ points [20], [32]:

(1) $$H_{K/K^{\prime }}=\pm v_{D}\left(\sigma_{x}\delta k_{x}+\sigma_{y}\delta k_{y}\right)\pm \gamma \sigma_{z},$$

where *v*_*D*_ is the group velocity, *σ*_*x*,*y*,*z*_ are the Pauli matrices, $\delta \vec{k}=\left(\delta k_{x},\delta k_{y}\right)$ is the reciprocal vector with respect to the *K*/*K*′ points, and *γ* denotes the strength of the symmetry-breaking perturbation (More details can be found in Appendix A). The valley Chern numbers are given by $C_{K/K^{\prime }}=\frac{1}{2\pi }\int_{HBZ_{K/K^{\prime }}}\Omega \left(\vec{k}\right)d^{2}k=\pm \mathrm{sgn}\left(\gamma \right)/2$ , where $\Omega \left(\vec{k}\right)$ is the Berry curvature, and the integration is carried over half of the first Brillouin zone (HBZ) around the *K*/*K*′ points [20], [23]. Since *γ* < 0 for VPC1 and *γ* > 0 for VPC2, the difference of the valley Chern number across the domain wall is $\left|\Delta C_{K/K^{\prime }}\right|=\left|C_{K/K^{\prime }}^{\text{VPC}1}-C^{\text{VPC}2}\right|=1$ , which is the nontrivial topological invariant that indicates the existence of topological edge states. We also calculated the Berry curvature using the fully numerical PWE method. The distribution of the normalized Berry curvature for the lowest band in VPC2 is plotted in Figure 1(d). It exhibits the *C*_3v_ symmetry which is different from the rotational symmetry of the Berry curvature distribution derived analytically from Equation (1). Nevertheless, nonzero Berry curvatures with opposite signs can be observed in Figure 1(d) to be highly localized around the *K* and *K*′ points, indicating the valley Chern numbers of opposite signs for different valleys. It is worth noting that the sign of the Berry curvature distribution is reversed for VPC1 as compared to that shown in Figure 1(d). According to the bulk-boundary correspondence, the nonzero difference between the valley Chern numbers of VPC1 and VPC2 guarantees that topological edge states exist at the interface in the real space and at the *K* and *K*′ valleys in the momentum space. In Figure 1(e), we present the *H*_*z*_ field distribution of the edge mode in one period along the *x* direction of the VPC structure shown in Figure 1(a). The mode profile is symmetric with respect to the domain wall (*y* = 0), and therefore it is often referred to as the even edge mode. The mode can be expressed by the Jackiw–Rebbi solution near the *K*/*K*′ points [32], [33], [34]:

(2) $$\left|\Psi_{K/K^{\prime }}^{\text{even}}\right\rangle =\frac{1}{\sqrt{2}}\left(\left|LCP\right\rangle +\left|RCP\right\rangle \right)e^{\pm i\delta k_{x}x}e^{\frac{\gamma }{v_{D}}\left|y\right|},$$

where $\left|LCP\right\rangle$ and $\left|RCP\right\rangle$ are the LCP and RCP Bloch modes at the *K*/*K*′ points on the two lowest bands of the unperturbed VPCs (*d*_1_ = *d*_2_), respectively. Note that *γ* < 0 in this case, which means the mode shows the characteristics of an evanescent wave along the *y* direction (More details can be found in Appendix A). Hereafter, we choose to build the topological photonic ADF based on the even mode because it can support low-loss propagation and has a smaller coupling loss with the fundamental mode in a silicon stripe waveguide, as we can see later. Figure 1(f) depicts the band structure for the PC with an interface which is illustrated in Figure 1(a). It reveals the presence of an edge state (blue) in the band gap between the first and second bulk bands (black). The edge state has almost linear dispersion near the *K* and *K*′ points, which is promising for achieving a large operation bandwidth. As the band structure is symmetric with respect to the wave vector *k*_*x*_, the group velocities at different valleys are constants of opposite signs, reflecting the preserved time reversal symmetry. It agrees well with the conclusion deduced from Equation (2) that the group velocities are ∓*v*_*D*_ for the *K*/*K*′ valleys, respectively (More details can be found in Appendix A).

### 2.2 Design of the topological photonic ADF

**Figure 2:**
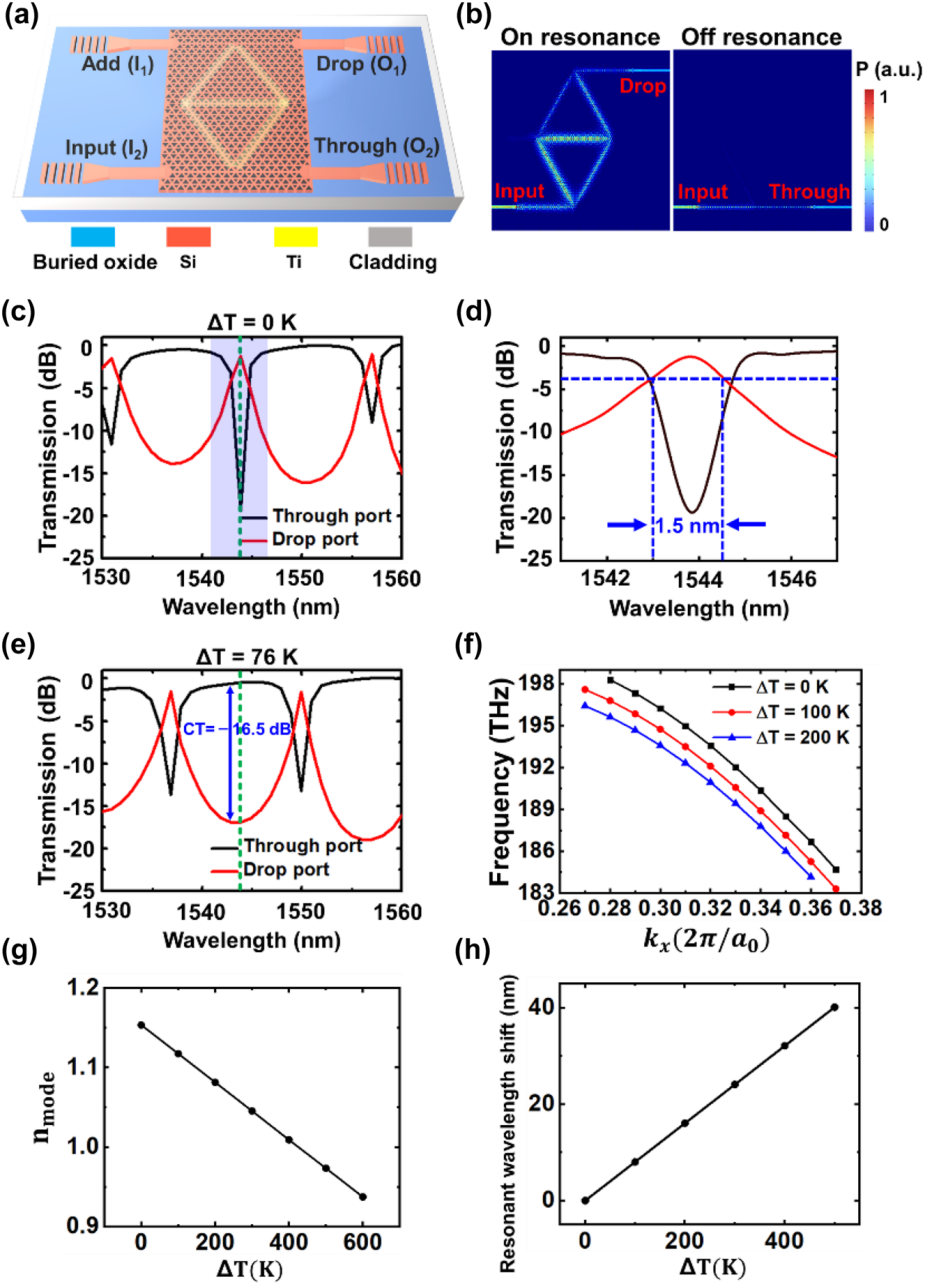
Schematic and simulation results of the topological photonic ADF. (a) Schematic of the proposed device. The light is coupled into and out of the VPC via grating couplers followed by 900-nm-wide silicon stripe waveguides. 2-μm-wide titanium stripes are deposited on top of the diamond-shaped microloop resonator to form the microheater. The add, drop, input and through ports are labelled as I_1_, O_1_, I_2_ and O_2_, respectively. (b) Simulated propagation profiles for the device when the light is injected from the input port. The light is output from the drop (through) port when the microloop resonator is on (off) resonance. (c) Simulated transmission spectra of the ADF without the thermal tuning. The green dashed line indicates the position of the working wavelength of 1,543.9 nm. (d) Zoom-in spectra of the area shaded in purple in (c). (e) Simulated transmission spectra of the ADF with the thermal tuning. The green dashed line indicates the position of the working wavelength of 1,543.9 nm. (f) Temperature dependence of the dispersion of the topological edge state. (g) Mode effective index of the topological edge state at 1,550 nm as a function of temperature rise. (h) Shift of the resonant wavelengths of the ADF as a function of temperature rise.

Based on the VPC structures and the edge states discussed above, we propose a compact and thermally tunable topological photonic ADF, as schematically illustrated in Figure 2(a). The ADF consists of a diamond-shaped microloop resonator with a side length of 15.6 μm side-coupled to two straight waveguides in the VPC. They are all formed by the domain walls that support the even edge mode which is topologically protected and therefore enables robust optical transport against sharp corners. Benefitting from this property, a compact device footprint of 17.4 × 28.2 μm^2^ is reached with a relatively low propagation loss and the device performance is almost intact in the presence of imperfections such as defects and dislocations (More details can be found in Appendix B). Considering the efficient coupling between the even edge mode and the fundamental mode in the stripe waveguide, 900-nm-wide silicon stripe waveguides are employed to connect the VPC straight waveguides with the grating couplers. A microheater composed of 2-μm-wide titanium stripes is placed on top of the microloop resonator for thermal tuning. To prove the feasibility of the proposed device, the full-wave numerical simulations were carried out using the 3D finite-difference time-domain (FDTD) methods. Figure 2(b) presents the propagation profiles for the device when the light is launched from the input (I_2_) port. At the resonant wavelengths, the light enters the microloop resonator and outputs from the drop (O_1_) port. For non-resonant wavelengths, however, the light passes through the bus waveguide to the through (O_2_) port without coupling into the microloop resonator. The transmission spectra with and without the thermal tuning are displayed in Figure 2(c) and (e), respectively. At the room temperature, the light at a resonant wavelength of 1,543.9 nm (indicated by the green dashed lines in Figure 2(c) and (e)) is output from the drop port, as shown in Figure 2(c). The 3-dB bandwidth of the resonant peak is ∼1.5 nm, as indicated in the zoom-in transmission spectra in Figure 2(d). It can be further expanded by cascading multiple microloop resonators to form a high-order filter, just like the flat-top filters based on cascaded microring resonators [35], [36]. When the temperature rises due to the microheater, the refractive index of silicon is changed, leading to the variation in the effective index of the edge state. The shift in the dispersion and the change in the mode effective index of the edge state are shown in Figure 2(f) and (g) as functions of rising temperature, respectively. Thanks to the high confinement of light enabled by VPCs and therefore the efficient interaction of light with the thermal field, a temperature increment of 76 K is sufficient to red-shift the resonant wavelength from 1,543.9 nm to 1,550 nm, as illustrated in Figure 2(e). The light at 1,543.9 nm is then output from the through port, implementing the thermal switching of the optical power from one output port to the other. It is worth mentioning that the change rate of the resonant wavelength shift versus the temperature rise is ∼0.08 nm/K, as shown in Figure 2(h). Therefore, precise temperature control is not necessary in practical applications of the proposed device to compensate for the ambient temperature variation. The simulation results show that the IL of the ADF is as low as 1.2 dB and the crosstalk (CT) is below −16.5 dB at the working wavelength of 1,543.9 nm, unveiling the potential for realizing high-performance functional devices with both the topological protection and the thermo-optic tunability.

### 2.3 Experimental demonstration of the topological photonic ADF

**Figure 3:**
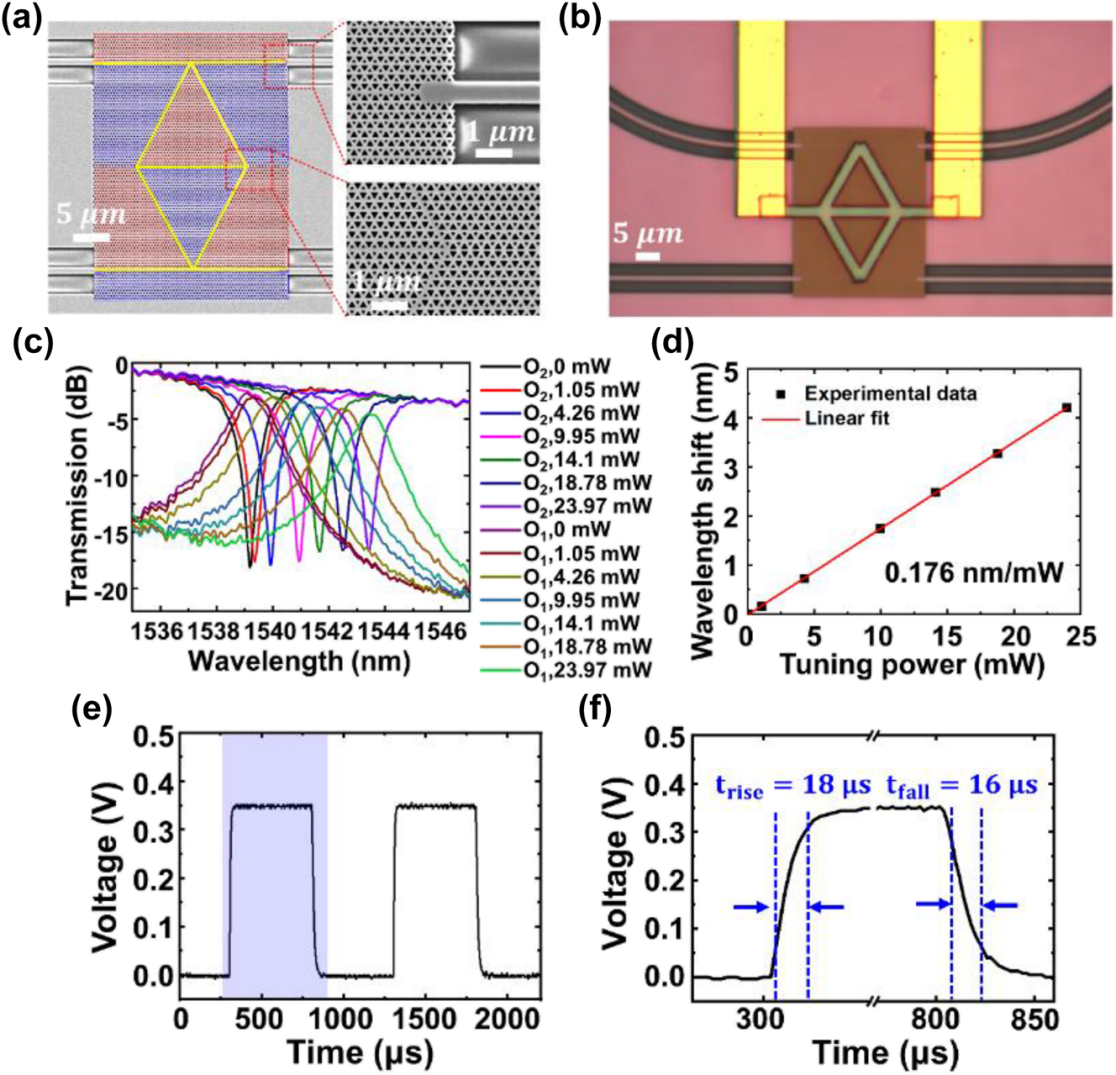
Experimental demonstration of the topological photonic ADF. (a) SEM images of the fabricated device. The VPC1 and VPC2 areas are shaded in red and blue, respectively, with the interface highlighted in yellow. The zoom-in SEM images of the VPC areas encircled by the red dashed boxes are shown in the insets. (b) Optical microscope photo of the fabricated device. The grey and golden stripes are the titanium microheater and the electrical wires linked to the contact pads, respectively. (c) Measured transmission spectra at the through and drop ports of the ADF with various tuning powers. (d) Fitting curve of the resonant wavelength shift as a function of tuning power. (e) Temporal response of the tunable ADF when a 1 kHz square-wave electrical signal is applied to the microheater. (f) Zoom-in figure of the rising edge and the falling edge of the signal period shaded in purple in (e).

We fabricated the proposed ADF on the SOI platform using E-beam lithography (EBL, Vistec EBPG 5200^+^), inductively coupled plasma (ICP, SPTS DRIE-I) etching, plasma enhanced chemical vapor deposition (PECVD, Oxford Plasmalab System 100) and E-beam evaporation. The fabrication process is totally complementary metal-oxide-semiconductor (CMOS)-compatible. Figure 3(a) shows the scanning electron microscope (SEM, Zeiss Ultra Plus) images of the VPC structure. The VPC1 and VPC2 areas are shaded in red and blue, respectively, with the interface highlighted in yellow. The zoom-in SEM images of the VPC areas encircled by the red dashed boxes are shown in the insets. A PC line defect is introduced at the input/output port to reduce the coupling loss between the VPC and the silicon stripe waveguide, as displayed in the top right inset. Figure 3(b) presents the optical microscope picture of the thermally tunable ADF. The grey and golden stripes are the titanium microheater and the electrical wires linked to the contact pads, respectively. Titanium was chosen as the microheater material for its high resistivity and melting point, which means it can generate heat more efficiently and reach higher temperature. The width and the thickness of the titanium stripes are 2 μm and 100 nm respectively, which are optimized to provide a high resistance and a proper overlap between the light and thermal fields. In the measurements, TE-polarized light from a tunable laser (Keysight 81960A) was coupled into and out of the chip by grating couplers. An optical power meter and a photodetector (PD, Keysight 81636B) were used for optical calibration and receiving the transmitted power, respectively. A voltage-current source-meter (Keithley 2,400) was employed to provide the electrical power required for thermal tuning. Figure 3(c) plots the measured transmission spectra at the through (O_2_) and drop (O_1_) ports of the ADF with different heating powers, which are all normalized to that of a reference grating coupler pair fabricated on the same wafer. The resonant wavelengths in the absence of thermal tuning are blue-shifted compared to the simulation results, which could be attributed to the inevitable fabrication errors. A clear red shift of the resonant wavelength near 1,540 nm can be observed with increasing heating power. When an electrical power of 23.97 mW is applied, the optical power at 1,539.2 nm is switched from the drop port to the through port. Thanks to the small size of the microloop resonator and the relatively slow group velocity of the topological edge mode, the free spectral range (FSR) of the ADF is 13.4 nm which is much larger than those of conventional MRRs. The Vernier effect can be exploited to further increase the FSR, which will certainly benefit its application in wavelength-division-multiplexing (WDM) systems [37], [38]. The measured IL is less than 2.7 dB and the CT is lower than −15.4 dB at this wavelength in both circumstances with and without the thermal tuning. The IL mainly originates from the coupling loss between the silicon stripe waveguide and the VPC straight waveguide. As one can see, it becomes larger with increasing heating power because the rising temperature changes the refractive index of silicon and therefore the coupling efficiency at the interfaces between the silicon stripe waveguide and the VPC straight waveguide. This issue can be addressed by moving the input/output ports away from the heating area. Therefore, we choose a proper device footprint of 17.4 × 28.2 μm^2^ in order to achieve a large FSR and low thermal crosstalk. The 3-dB bandwidth of the resonant peak/notch is ∼1.5 nm, corresponding to a loaded quality (Q) factor over 10^3^ for the microloop resonator. The 3-dB bandwidth can be further reduced by increasing the thickness of the silicon slab to suppress the out-of-plane radiation of light. The tuning efficiency of the ADF is measured to be 0.176 nm/mW, as illustrated in Figure 3(d). It can be improved by reducing the thickness of the silica cladding layer to make the heat transfer through the cladding layer more efficient. To find out the response time of the tunable device, a 1-kHz square-wave electrical signal generated by the arbitrary waveform generator (AWG, Rigol DG4202) was applied to the microheater. The optical power at the drop port was monitored using a high-speed PD (Finisar XPDV2120R) whose output electrical signal was recorded and displayed using a digital storage oscilloscope (DSO, Tektronix TBS 1102), as shown in Figure 3(e). The rising edge and the falling edge of the signal period shaded in purple in Figure 3(e) are plotted in Figure 3(f). The 10 %–90 % switching time constants are 18 μs and 16 μs for the rising edge and the falling edge, respectively.

### 2.4 High-speed data transmission experiment

**Figure 4:**
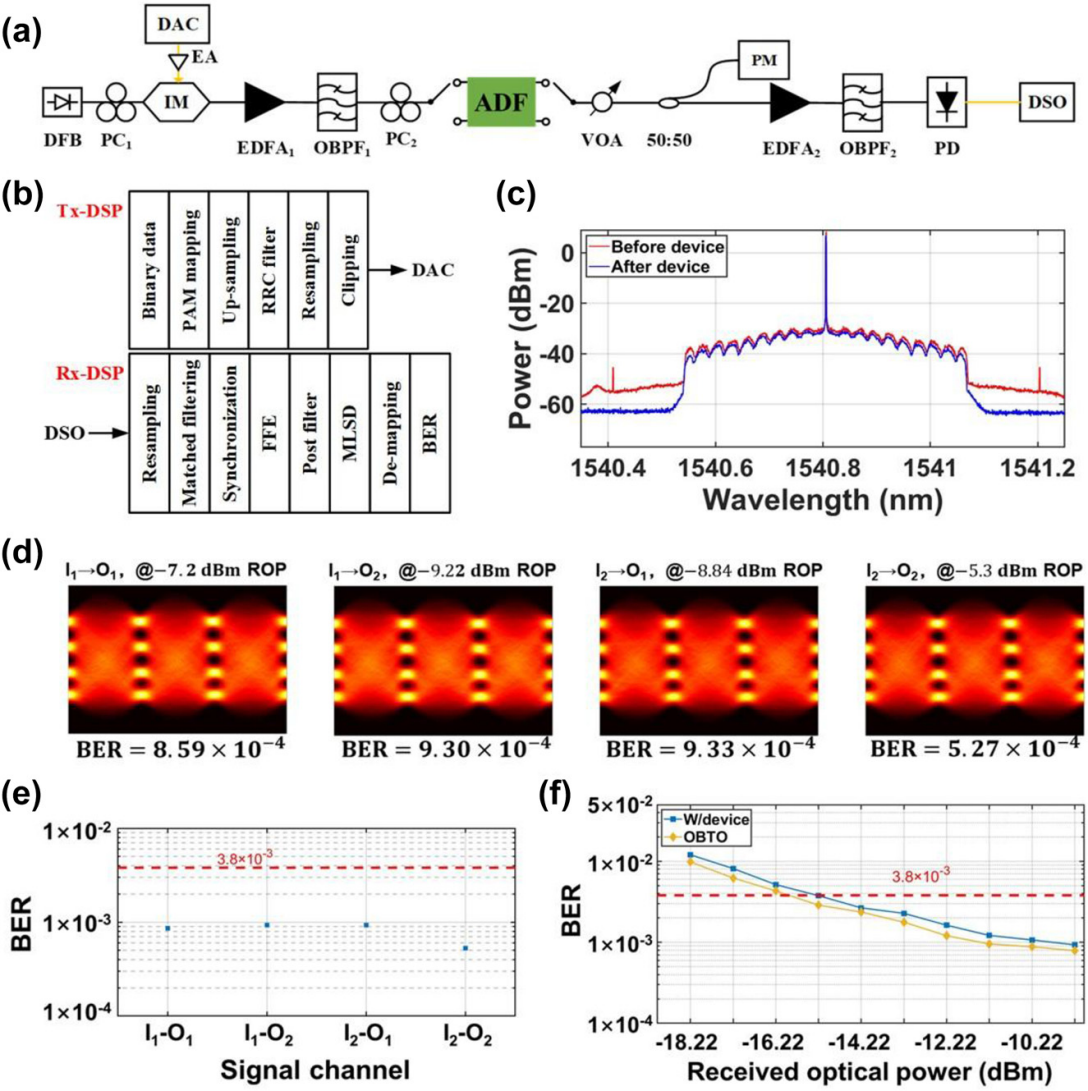
High-speed data transmission experiment based on the topological photonic ADF. (a) Experimental setup for the high-data-rate transmission experiment. The black and yellow lines represent optical and electrical links, respectively. OBPF, optical bandpass filter; PM, power meter. (b) DSP flow charts for the transmitter and the receiver. (c) Optical spectra of the modulated signals before and after passing the device. (d) Eye diagrams for the four switching configurations. ROP, received optical power. (e) BERs for the four signal channels. (f) BER versus received optical power for the worst switching state (I_1_–O_2_).

To explore the application of this topological photonic device in a practical optical communication system, we performed a high-speed data transmission experiment using a 66-GBaud PAM-4 signal. The experimental setup and the transceiver digital signal processing (DSP) flow charts are shown in Figure 4(a) and (b), respectively. Figure 4(c) shows the optical spectra of the modulated signals before and after passing the device which are measured by an optical spectrum analyzer (OSA) with a high resolution of 1.12 pm. The eye diagrams of the recovered PAM-4 signals with different switching configurations (I_1_–O_1_, I_1_–O_2_, I_2_–O_1_, I_2_–O_2_) are provided in Figure 4(d) while the bit error rates (BERs) are calculated and given in Figure 4(e). The 7 % hard-decision forward error correction (FEC) threshold of 3.8 × 10^−3^ is achieved for all the switching configurations. We also measured the BER curves for the worst switching state (I_1_–O_2_), as shown in Figure 4(f). Compared to the OBTB sensitivity of −16 dBm, a low penalty of <0.7 dB is observed for the transmitted signal in the presence of the device, indicating that the performance of the proposed device is good enough for the high-capacity transmission at a raw data rate of 132 Gb/s. Given the 7 % FEC overhead and the frame redundancy, the 66-GBaud PAM-4 signal has a net data rate of 119.77 Gb/s.

Table 1 compares the performance of various tunable silicon photonic devices based on traditional structures such as MRRs, MZIs and nanobeams and topological PCs as well. The proposed ADF based on VPCs has a very low IL and an ultrahigh data rate compared to the different kinds of devices listed in this table. Combined with its small footprint, low switching power and moderate CT value, our design shows great promise for the application in on-chip optical communications where both the topological protection and the thermal tunability are very much desired for the transmission and switching of data. For example, in the presence of scatters such as defects and dislocations the transmission spectra of the VPC-based devices are almost unchanged compared to the unperturbed structure (More details can be found in Appendix B). In contrast, for conventional devices based on structures such as MRRs the scatters will inevitably introduce extra losses and probably cause mode splitting that completely alters the transmission spectra.

**Table 1:** Comparison of various tunable silicon photonic devices.

Reference	Structure	Tuning method	Footprint [μm^2^]	IL [dB]	CT [dB]	Switching power [mW]	Date rate [Gb/s]
[39]	MRR	Electro-optic effect	40 × 20	5.9	−50	37	30
[40]	MRR	Thermo-optic effect	40 × 20	4.9	–	21	25
[41]	MZI	Thermo-optic effect	4.6 × 1 mm^2^	3.7	−7.2	104.8	25
[42]	Nanobeam	Thermo-optic effect	60 × 16	4.4	−13.5	0.15	124
[25]	VPC waveguide	Optical nonlinearity	15 × 40	–	−8.2	∼7.8 W	–
[27]	VPC MRR	Thermo-optic effect	20 × 12	15	−25	24	25
[26]	VPC switch	Thermo-optic effect	25.7 × 28.3	6.2	−14	18.2	132
This work	VPC ADF	Thermo-optic effect	17.4 × 28.2	2.7	−15.4	23.97	132

## 3 Conclusions

In conclusion, we have proposed and experimentally demonstrated a thermally tunable topological photonic ADF based on VPCs. By exploiting the topologically protected edge state and therefore the robust transport of light against sharp bends, a compact footprint of 17.4 × 28.2 μm^2^ and a low IL of <2.7 dB are achieved for the proposed device at the working wavelength. The device footprint can be further reduced by employing new techniques such as cladding-free waveguides [43], [44] and epsilon-near-zero claddings [45] in the VPC design. A diamond-shaped microloop resonator is employed to confine the light and enhance its interaction with the thermal field. As a result, a low electrical power of only 23.97 mW is needed to switch the optical power between two output ports through the thermal tuning. In addition, a high-speed data transmission experiment based on the device was performed with a 66-GBaud PAM-4 signal. The sensitivity penalty induced by the ADF is no more than 0.7 dB compared with the OBTB case, manifesting its capability of supporting on-chip telecommunications at ultrahigh data rates. Our work proves the feasibility of realizing high-performance tunable devices with silicon-based VPCs, inspiring further investigations into topological photonics for practical applications such as optical communications, nanophotonics and quantum information processing.

## Figures and Tables

**Figure B.1:**
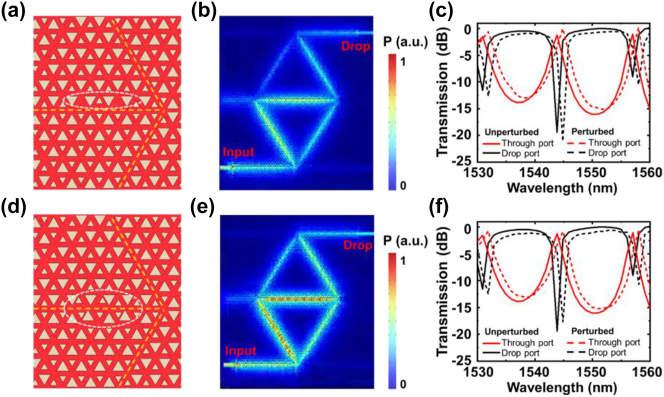
Robustness analysis of the proposed device. (a) Schematic of the defect formed by randomly missing two triangular holes and dislocating one triangular hole at the resonator interface. (b) Simulated propagation profile in the presence of the defect when the resonator is on resonance. (c) Simulated transmission spectra of the ADF in the presence of the defect shown in (a). (d) Schematic of the dislocation formed by randomly translating four triangular holes at the resonator interface. (e) Simulated propagation profile in the presence of the dislocation when the resonator is on resonance. (f) Simulated transmission spectra of the ADF in the presence of the dislocation shown in (d).

## Appendix A: Effective Hamiltonian derivation and analytical expression of the edge state

In this section, we present the analytical derivation of the effective Hamiltonian describing the band diagrams of the valley photonic crystals (VPCs) near the *K* and *K*′ points and the analytical expression of the topological edge state. First, we consider a two-dimensional (2D) VPC with a *C*_6v_ symmetry (*d*_1_ = *d*_2_) which is schematically illustrated in Figure 1(a) of the main text. By using the plane wave expansion (PWE) method, we transform the electromagnetic wave equations to an eigen value problem governed by the effective Hamiltonian. The Helmholtz equation for the transverse electric (TE) mode (*H*_*z*_) supported by the VPCs can be written as [20], [26]:

(A1)
$${\left(\frac{\omega }{c}\right)}^{2}H_{z}+\frac{\partial }{\partial x}\left(\frac{1}{\varepsilon }\frac{\partial }{\partial x}H_{z}\right)+\frac{\partial }{\partial y}\left(\frac{1}{\varepsilon }\frac{\partial }{\partial y}H_{z}\right)=0,$$

where *ω* is the eigen frequency of the Bloch mode, and *c* is the speed of light in vacuum. Since the VPCs are periodic in structure, we can expand the magnetic field *H*_*z*_ and the inverse permittivity *ε* ^−1^ as follows:

(A2)
$$H_{z}\left(\vec{r},\vec{q}\right)=\underset{\vec{G}}{\sum }H_{\vec{G}}{\text{e}}^{\text{i}\left(\vec{q}+\vec{G}\right)\cdot \vec{r}},$$

(A3)
$$\varepsilon^{-1}\left(\vec{r}\right)=\underset{\vec{G}}{\sum }\beta_{\vec{G}}{\text{e}}^{\text{i}\vec{G}\cdot \vec{r}},$$

where $\vec{r}$ is the position vector, $\vec{q}$ is the wave vector, and $\vec{G}$ represent the reciprocal lattice vectors. Substituting Equations (A2) and (A3) into Equation (A1) yields:

(A4)
$${\left(\frac{\omega }{c}\right)}^{2}H_{\vec{G}}=\underset{{\vec{G}}^{\prime }}{\sum }\beta_{\vec{G}-{\vec{G}}^{\prime }}\left[\left(\vec{q}+\vec{G}\right)\cdot \left(\vec{q}+{\vec{G}}^{\prime }\right)\right]H_{{\vec{G}}^{\prime }}.$$

Since we are interested in the band structure in the vicinity of the *K* point, we consider the waves propagating with wave vectors $\vec{q}=\vec{K}+\vec{\delta k}$ , where $\vec{\delta k}$ is the small perturbation of the wave vector with respect to that of the *K* point $\vec{K}=\left[4\pi /3a_{0},0\right]$ . We also limit the PWE to the plane waves with three shortest reciprocal vectors ${\vec{G}}_{0}=\left[0,0\right]$ , ${\vec{G}}_{1}=\left[-a_{0}/2\pi ,-\sqrt{3}/2\right]$ and ${\vec{G}}_{2}=\left[-a_{0}/2\pi ,\sqrt{3}/2\right]$ . Then Equation (A4) can be simplified as [20], [26]:

(A5)
$${\left(\frac{\omega }{c}\right)}^{2}\tilde{H}=M\tilde{H},$$

where $M=\frac{K_{x}^{2}}{2}\left[\begin{array}{ccc} 2\beta_{0} & -\beta_{G} & -\beta_{G}\\ -\beta_{G} & 2\beta_{0} & -\beta_{G}\\ -\beta_{G} & -\beta_{G} & 2\beta_{0} \end{array}\right]+K_{x}\delta k_{x}\left[\begin{array}{ccc} 2\beta_{0} & \beta_{G}/2 & \beta_{G}/2\\ \beta_{G}/2 & -\beta_{0} & -\beta_{G}\\ \beta_{G}/2 & -\beta_{G} & -\beta_{0} \end{array}\right]+\frac{\sqrt{3}K_{x}\delta k_{y}}{2}\left[\begin{array}{ccc} 0 & \beta_{G} & -\beta_{G}\\ \beta_{G} & 2\beta_{0} & 0\\ -\beta_{G} & 0 & -2\beta_{0} \end{array}\right]$ and $\tilde{H}={\left[\begin{array}{ccc} H_{\vec{G_{0}}} & H_{\vec{G_{1}}} & H_{\vec{G_{2}}} \end{array}\right]}^{\text{T}}$ . Here, we denote $\beta_{\vec{G_{0}}}=\beta_{0}$ and $\beta_{\vec{G_{1}}}=\beta_{\vec{G_{2}}}=\beta_{G}$ . By applying the linear transformation $\tilde{U}=\frac{1}{\sqrt{3}}\left[\begin{array}{ccc} 1 & 1 & 1\\ 1 & \eta^{2} & \eta \\ 1 & \eta & \eta^{2} \end{array}\right]$ with $\eta =\exp\left(i\frac{2\pi }{3}\right)$ and neglecting the singlet state, Equation (A5) can be rewritten as [20], [26]:

(A6)
$${\left(\frac{\omega }{c}\right)}^{2}{\tilde{H}}_{d}=\left[\begin{array}{cc} \left(\beta_{0}+\beta_{G}/2\right)K_{x}^{2} & \left(\beta_{0}-\beta_{G}\right)\left(\delta k_{x}-i\delta k_{y}\right)K_{x}\\ \left(\beta_{0}-\beta_{G}\right)\left(\delta k_{x}+i\delta k_{y}\right)K_{x} & \left(\beta_{0}+\beta_{G}/2\right)K_{x}^{2} \end{array}\right]{\tilde{H}}_{d},$$

where ${\tilde{H}}_{d}={\left[\begin{array}{cc} H_{\text{RCP}} & H_{\text{LCP}} \end{array}\right]}^{T}$ are the right- and left-handed circular polarized doublet states with $H_{\text{RCP}}=\frac{1}{\sqrt{3}}\left(H_{\vec{G_{0}}}+\eta^{2}H_{\vec{G_{1}}}+\eta H_{\vec{G_{2}}}\right)$ and $H_{\text{RCP}}=\frac{1}{\sqrt{3}}\left(H_{\vec{G_{0}}}+\eta^{2}H_{\vec{G_{1}}}+\eta H_{\vec{G_{2}}}\right)$ . Then the effective Hamiltonian of the VPC with a *C*_6v_ symmetry (*d*_1_ = *d*_2_) can be expressed as $H_{K}=v_{D}\left(\sigma_{x}\delta k_{x}+\sigma_{y}\delta k_{y}\right)$ near the *K* point, where *v*_*D*_ is the group velocity, and *σ*_*x*,*y*_ are the Pauli-*x* and Pauli-*y* matrices. Here, we define $v_{D}=\left(\beta_{0}-\beta_{G}\right)\left(\delta k_{x}-i\delta k_{y}\right)K_{x}$ and set the eigen value $\left(\beta_{0}+\beta_{G}/2\right)K_{x}^{2}$ at the Dirac point to zero.

When the *C*_6v_ symmetry is broken by introducing asymmetry between two holes within the unit cell (*d*_1_ ≠ *d*_2_), the Hamiltonian becomes:

(A7)
$$H_{K}=v_{D}\left(\sigma_{x}\delta k_{x}+\sigma_{y}\delta k_{y}\right)+\gamma \sigma_{z},$$

where *σ*_*z*_ is the Pauli-*z* matrix, and *γ* denotes the strength of the symmetry-breaking perturbation. *γ* can be calculated by *γ* = −∫Δ*ɛE***E*d*V*, where Δ*ɛ* is the permittivity perturbation, *E* is the electric field distribution of the RCP mode, and the integration runs over the volume of the unit cell. Following a similar deduction, one can obtain the effective Hamiltonian at the *K*′ valley as well:

(A8)
$$H_{K^{\prime }}=-v_{D}\left(\sigma_{x}\delta k_{x}+\sigma_{y}\delta k_{y}\right)-\gamma \sigma_{z}.$$

Remember that *γ* < 0 in this case, so one can derive the eigen states in the proximity of the *K* point according to Equation (A7):

(A9)
$$\begin{array}{cc} \left|\psi_{+}\right\rangle & =\left[\begin{array}{c} \frac{v_{D}\delta k_{x}-iv_{D}\delta k_{y}}{\sqrt{v_{D}^{2}\left(\delta k_{x}^{2}+\delta k_{y}^{2}\right)+\gamma^{2}}-\gamma }\\ 1 \end{array}\right],\\ \left|\psi_{-}\right\rangle & =\left[\begin{array}{c} 1\\ \frac{v_{D}\delta k_{x}+iv_{D}\delta k_{y}}{\sqrt{v_{D}^{2}\left(\delta k_{x}^{2}+\delta k_{y}^{2}\right)+\gamma^{2}}-\gamma } \end{array}\right], \end{array}$$

which correspond to the eigen values $\omega_{\pm }=\pm \sqrt{v_{D}^{2}\left(\delta k_{x}^{2}+\delta k_{y}^{2}\right)+\gamma^{2}}$ , respectively. Therefore, the Bloch modes on the upper and lower bands at the *K* point (*δk*_*x*_ = *δk*_*y*_ = 0) would be $\left|\psi_{+}\right\rangle =\left|LCP\right\rangle$ and $\left|\psi_{-}\right\rangle =\left|RCP\right\rangle$ , as indicated in Figure 1(b) and (c) of the main text.

For the interface formed by two types of VPCs shown in Figure 1(a) of the main text, the effective Hamiltonian can be written as [23], [32]:

(A10)
$$\begin{array}{cc} H_{K/K^{\prime }} & =\pm v_{D}\left(\sigma_{x}\delta k_{x}+\sigma_{y}\delta k_{y}\right)\pm m\left(y\right)\sigma_{z}\\ & =\mp iv_{D}\left(\sigma_{x}\partial_{x}+\sigma_{y}\partial_{y}\right)\pm m\left(y\right)\sigma_{z}, \end{array}$$

where $m\left(y\right)=\left\{\begin{array}{c} \gamma ,y>0\\ 0,y=0\\ -\gamma ,y<0 \end{array}\right.$ describes the potential of opposite signs across the domain wall at *y* = 0. The edge state can be expressed analytically by the Jackiw–Rebbi solution at the *K* and *K*′ valleys, which reads [33], [34]:

(A11)
$$\begin{array}{cc} \left|\psi_{K/K^{\prime }}^{\text{even}}\right\rangle & =\frac{1}{\sqrt{2}}\left[\begin{array}{c} 1\\ 1 \end{array}\right]e^{\pm i\delta k_{x}x}e^{\overset{y}{\underset{0}{\int }}\frac{1}{v_{D}}m\left(y^{\prime }\right)dy^{\prime }}\\ & =\frac{1}{\sqrt{2}}\left[\begin{array}{c} 1\\ 1 \end{array}\right]e^{\pm i\delta k_{x}x}e^{\frac{\gamma }{v_{D}}\left|y\right|}. \end{array}$$

The mode shows the behavior of a propagating wave along the *x* direction and the characteristics of an evanescent wave along the *y* direction as *γ* < 0. Its mode profile is also symmetric with respect to the domain wall because the part before the exponential terms in Equation (A11) represents a superposition of the LCP and RCP states. Therefore, it is often referred to as the even edge mode. Most importantly, the eigen value corresponding to the Jackiw–Rebbi solution is *ω*_*K*/*K*′_ = ∓*v*_*D*_ *δk*_*x*_, which means the group velocities at the *K*/*K*′ valleys are ∓*v*_*D*_ for the edge mode.

## Appendix B: Robustness of the topological photonic ADF

To demonstrate the robustness of the topological photonic ADF against fabrication imperfections, we consider two kinds of errors that could be introduced during the fabrication process. The first one is a defect formed by missing two triangular holes and dislocating one triangular hole at the resonator interface, as shown in Figure B.1(a). Again, the propagation profile and the transmission spectra in the presence of the defect are obtained using full 3D FDTD simulations. The results are presented in Figure B.1(b) and (c), respectively. No obvious scattering is observed at the defect location and the perturbed transmission spectra (with defect) is almost identical to those of the unperturbed structure (without defect) other than a slight shift in resonant wavelength. The second type of fabrication imperfection is the dislocation of four triangular holes at the resonator interface, which is illustrated in Figure B.1(d). The simulated propagation profile and transmission spectra in this case are plotted in Figure B.1(e) and (f). As one can see, the dislocation has a minor effect on the propagation of the edge mode and therefore barely changes the transmission spectra at the through and drop ports of the proposed device (other than a slight shift in resonant wavelength).

## References

[1] Qi X.-L., Zhang S. C. (2011). Topological insulators and superconductors. *Rev. Mod. Phys.*.

[2] Hasan M. Z., Kane C. L. (2010). Colloquium: topological insulators. *Rev. Mod. Phys.*.

[3] Bernevig B. A., Hughes T. L. (2013). *Topological Insulators and Topological Superconductors*.

[4] Lu L., Joannopoulos J. D., Soljačić M. (2014). Topological photonics. *Nat. Photonics*.

[5] Khanikaev A. B., Shvets G. (2017). Two-dimensional topological photonics. *Nat. Photonics*.

[6] Ozawa T. (2019). Topological photonics. *Rev. Mod. Phys.*.

[7] Susstrunk R., Huber S. D. (2015). Observation of phononic helical edge states in a mechanical topological insulator. *Science*.

[8] Lu J. (2017). Observation of topological valley transport of sound in sonic crystals. *Nat. Phys.*.

[9] Nash L. M., Kleckner D., Read A., Vitelli V., Turner A. M., Irvine W. T. M. (2015). Topological mechanics of gyroscopic metamaterials. *Proc. Natl. Acad. Sci. U. S. A.*.

[10] Huber S. D. (2016). Topological mechanics. *Nat. Phys.*.

[11] Wu Y., Li C., Hu X., Ao Y., Zhao Y., Gong Q. (2017). Applications of topological photonics in integrated photonic devices. *Adv. Opt. Mater.*.

[12] Wang Z., Chong Y. D., Joannopoulos J. D., Soljačić M. (2015). Reflection-free one-way edge modes in a gyromagnetic photonic crystal. *Phys. Rev. Lett.*.

[13] Wang Z., Chong Y. D., Joannopoulos J. D., Soljačić M. (2009). Observation of unidirectional backscattering-immune topological electromagnetic states. *Nature*.

[14] Khanikaev A. B., Mousavi S. H., Tse W.-K., Kargarian M., MacDonald A. H., Shvets G. (2013). Photonic topological insulators. *Nat. Mater.*.

[15] Chen W.-J. (2014). Experimental realization of photonic topological insulator in a uniaxial metacrystal waveguide. *Nat. Commun.*.

[16] Rechtsman M. C. (2013). Photonic Floquet topological insulators. *Nature*.

[17] Hafezi M., Mittal S., Fan J., Migdall A., Taylor J. M. (2013). Imaging topological edge states in silicon photonics. *Nat. Photonics*.

[18] Wu L.-H., Hu X. (2015). Scheme for achieving a topological photonic crystal by using dielectric material. *Phys. Rev. Lett.*.

[19] Barik S. (2018). A topological quantum optics interface. *Science*.

[20] Shalaev M. I., Walasik W., Tsukernik A., Xu Y., Litchinitser N. M. (2019). Robust topologically protected transport in photonic crystals at telecommunication wavelengths. *Nat. Nanotechnol.*.

[21] He X.-T. (2019). A silicon-on-insulator slab for topological valley transport. *Nat. Commun.*.

[22] Zeng Y. (2010). Electrically pumped topological laser with valley edge modes. *Nature*.

[23] Wang H. (2022). Asymmetric topological valley edge states on silicon-on-insulator platform. *Laser Photonics Rev.*.

[24] Cheng X., Jouvaud C., Ni X., Mousavi S. H., Genack A. Z., Khanikaev A. B. (2016). Robust reconfigurable electromagnetic pathways within a photonic topological insulator. *Nat. Mater.*.

[25] Shalaev M. I., Walasik W., Litchinitser N. M. (2019). Optically tunable topological photonic crystal. *Optica*.

[26] Wang H. (2022). Ultracompact topological photonic switch based on valley-vortex-enhanced high-efficiency phase shift. *Light: Sci. Appl.*.

[27] Qi Z. (2022). Electrical tunable topological valley photonic crystals for on-chip optical communications in the telecom band. *Nanophotonics*.

[28] Zhou Z., Yin B., Deng Q., Li X., Cui J. (2015). Lowering the energy consumption in silicon photonic devices and systems. *Photonics Res.*.

[29] Soref R. A., Bennett B. R. (1987). Electrooptical effects in silicon. *IEEE J. Quantum Electron.*.

[30] Reed G. T., Mashanovich G., Gardes F. Y., Thomson D. J. (2010). Silicon optical modulators. *Nat. Photonics*.

[31] Chen X.-D., Zhao F.-L., Chen M., Dong J.-W. (2017). Valley-contrasting physics in all-dielectric photonic crystals: orbital angular momentum and topological propagation. *Phys. Rev. B*.

[32] Zhang L. (2019). Valley kink states and topological channel intersections in substrate-integrated photonic circuitry. *Laser Photonics Rev.*.

[33] Shen S.-Q. (2012). *Topological Insulators: Dirac Equation in Condensed Matters*.

[34] Franz M., Molenkamp L. (2013). *Topological Insulators*.

[35] Xia F., Rooks M., Sekaric L., Vlasov Y. (2007). Ultra-compact high order ring resonator filters using submicron silicon photonic wires for on chip optical interconnects. *Opt. Express*.

[36] Chen P., Chen S., Guan X., Shi Y., Dai D. (2014). High-order microring resonators with bent couplers for a box-like filter response. *Opt. Lett.*.

[37] Griffel G. (2000). Valley kink states and topological channel intersections in substrate-integrated photonic circuitry. *IEEE Photonics Technol. Lett.*.

[38] Morichetti F. (2021). Polarization-transparent silicon photonic add-drop multiplexer with wideband hitless tuneability. *Nat. Commun.*.

[39] Luo X. (2012). Silicon high-order coupled-microring-based electro-optical switches for on-chip optical interconnects. *IEEE Photonics Technol. Lett.*.

[40] Dong P. (2010). Thermally tunable silicon racetrack resonators with ultralow tuning power. *Opt. Express*.

[41] Li Z. (2023). Ultra-low-loss multi-layer 8× 8 microring optical switch. *Photonics Res.*.

[42] Zhang R. (2020). Ultracompact and low-power-consumption silicon thermo-optic switch for high-speed data. *Nanophotonics*.

[43] Song T. (2022). Ultracompact photonic circuits without cladding layers. *Phys. Rev. X*.

[44] Ji W., Luo J., Song T., Peng R., Wang M., Lai Y. (2024). Cladding-free hyperbolic waveguide arrays. *ACS Photonics*.

[45] Ji W. (2022). Crosstalk prohibition at the deep-subwavelength scale by epsilon-near-zero claddings. *Nanophotonics*.

